# Assessing biological aging: the origin of deficit accumulation

**DOI:** 10.1007/s10522-013-9446-3

**Published:** 2013-07-17

**Authors:** Arnold Mitnitski, Xiaowei Song, Kenneth Rockwood

**Affiliations:** 1 Department of Medicine, Dalhousie University, Suite 229-5790 University Ave., Halifax, NS B3H 1V7 Canada; 2 Department of Mathematics and Statistics, Dalhousie University, Halifax, NS Canada; 3 Division of Geriatric Medicine, QEII Health Science Centre, Suite 1421-5955 Veterans’ Memorial Lane, Halifax, NS B3H 2E1 Canada

**Keywords:** Health status, Deficit accumulation, Frailty, Fitness-frailty continuum, Mortality, Stochastic process

## Abstract

The health of individuals is highly heterogeneous, as is the rate at which they age. To account for such heterogeneity, we have suggested that an individual’s health status can be represented by the number of health deficits (broadly defined by biological and clinical characteristics) that they accumulate. This allows health to be expressed in a single number: the frailty index (FI) is the ratio of the deficits present in a person to the total number of deficits considered (e.g. in a given database or experimental procedure). Changes in the FI characterize the rate of individual aging. The behavior of the FI is highly characteristic: it shows an age specific, nonlinear increase, (similar to Gompertz law), higher values in females, strong associations with adverse outcomes (e.g., mortality), and a universal limit to its increase (at FI ~0.7). These features have been demonstrated in dozens of studies. Even so, little is known about the origin of deficit accumulation. Here, we apply a stochastic dynamics framework to illustrate that the average number of deficits present in an individual is the product of the average intensity of the environmental stresses and the average recovery time. The age-associated increase in recovery time results in the accumulation of deficits. This not only explains why the number of deficits can be used to estimate individual differences in aging rates, but also suggests that targeting the recovery rate (e.g. by preventive or therapeutic interventions) will decrease the number of deficits that individuals accumulate and thereby benefit life expectancy.

**Electronic supplementary material:**

The online version of this article (doi:10.1007/s10522-013-9446-3) contains supplementary material, which is available to authorized users.

## Introduction

Aging is a complex phenomenon but even so shows remarkable regularities in such characteristics as survival probability and mortality rates. Although these regularities can be observed reliably at the population level (the most famous being the Gompertz law of mortality), they are masked at the individual level by inter-personal variability. Individuals age in a variety of ways, making attractive the idea that they differ in their biological age.

The estimation of biological age is difficult: how to integrate biological markers into such a measure of biological age is not clear. Recent attempts have been based on a battery of biological (performance) markers, used to construct an index of physical fitness age from 13 performance measures (Kimura et al. 2012). Many commentators find it attractive to assess biological aging using physiological measures that change with age; even so, the prospects for success with this approach are controversial (Ingram et al. 2001; Kulminski et al. 2009). There is some evidence however that a wide range of health related variables, and not just laboratory measures, can be used to assess biological age if they are combined appropriately. Such variables are readily available in large clinical and epidemiological databases (Mitnitski et al. 2005; Goggins et al. 2005; Yashin et al. 2007a, 2013; Gu et al. 2009; Theou et al. 2013). One such tool, the frailty index (FI), has been used to incorporate a range of health deficits (including biological ones) that people accumulate during their life course (Mitnitski et al. 2001; Kulminski et al. 2007; Yashin et al. 2007a).

While different individuals face a variety of different health problems, there is a common denominator—the number of health problems increases with aging and increases faster in those individuals whose health is poor (Yashin et al. 2007a; Kulminski et al. 2007). Ageing develops gradually and starts from small changes in health (Kirkwood 2005) which accumulate across the adult life course (Rockwood et al. 2011). While many of the variables, when considered in isolation from each other, have only small effects on health, their cumulative effect becomes significant (Mitnitski et al. 2001, 2002; Kulminski et al. 2007). These cumulative effects can be quantified by combining health related variables (either biological or clinical, including even those self-reported) in a so-called fitness/frailty index or more often just a FI. This term reflects its extensive use in medicine, where the increased vulnerability of older adults to adverse outcomes is referred to as frailty (Fulop et al. 2010; Rockwood and Mitnitski 2011; Clegg et al. 2013).

Such measures have been investigated in both epidemiological surveys and clinical databases (Jones et al. 2004; Mitnitski et al. 2005; Woo et al. 2006; Rockwood et al. 2007, 2011; Kulminski et al. 2006; Dupre et al. 2009; Yang and Lee 2010; Singh et al. 2012; Eeles et al. 2012). These indices have been shown to be useful indicators of ageing, good predictors of adverse outcomes such as worsening health (Mitnitski et al. 2006; Fallah et al. 2011), poor response to vaccination (Ridda et al. 2009), institutionalization (Jones et al. 2005; Rockwood et al. 2007) and death (Jones et al. 2005; Mitnitski et al. 2005; Kulminski et al. 2007; Yashin et al. 2007a; Eeles et al. 2012; Singh et al. 2012). Recently this approach has been extended to animal models (Parks et al. 2012).

Of some interest, the properties of the FIs depend more on the number of deficits from which the FIs are comprised rather than on their nature (Rockwood et al. 2006; Rockwood and Mitnitski 2011) reflecting that health related variables are rarely independent. Given this interdependence, the rate of increase in the accumulation of deficits has been proposed as an estimate of the rate of aging (Mitnitski et al. 2001). That has some support: in general, the FI characterises individual health across the fitness-frailty continuum from the fittest (those who compared to others at their age, have accumulated just a few health problems) to the frailest people who, having accumulated many more problems than have others of their age, are the most vulnerable to stresses. In consequence, the fit are less prone, and the frail are more prone to adverse outcomes such as major illnesses, disabilities and mortality. The behaviour of the FI is highly characteristic: it accumulates exponentially with age (resembling Gompertz law), is strongly associated with mortality and has a distribution that changes with age but still has a limit of about 0.7, beyond which further deficit accumulation is incompatible with life (Rockwood and Mitnitski 2006; Bennett et al. 2013).

The individual age trajectories of the FI show stochastic dynamic behaviour (Mitnitski et al. 2006, 2012; Fallah et al. 2011). Importantly, even though, on average, deficits accumulate over time, individual trajectories can either increase (indicating worsening of heath) or decrease. This indicates that health improvement is not just possible, but quite common, at least for a short run, likely reflecting medical interventions, changes in life styles, etc.

Despite the advances that have been made in understanding aging systems, the complexity of aging challenges researchers, especially those who are rooted in the success of the reductionist approach. Progress in considering multiple aging processes requires a systems biology approach (Jazwinski 2002; Tacutu et al. 2010a; Kirkwood 2011; Yashin et al. 2012). This is based on mathematical modeling, which makes it possible to apply mathematical insights and apparatus developed in other scientific fields, as commonly is done in other scientific areas, including physics, engineering, operations research etc.

Here, we consider a general stochastic model of how deficit accumulation results from interactions between two processes: the process of (broadly construed) *environmental stresses*, which cause damage in multiple systems of the organism, and that of *damage control and recovery*. Both processes are intrinsically stochastic, as there are always a multiplicity of factors that cannot be controlled. The model we employ here is based on a queuing theory approach (Erlang 1917; Gross and Harris 1998) and reflects a formal analogy between the number of deficits that the individuals have accumulated and the length of a queue in the queuing system such as a server. The model allows an understanding of the major patterns in deficits accumulation observed in many studies by our group (e.g., Mitnitski et al. 2001, 2005; Rockwood and Mitnitski 2006; Song et al. 2010) and others (García-González et al. 2009; Yang and Lee 2010). In particular, we show that the age-associated decline in the recovery rate (or the associated increase in the average time of recovery) together with the stochastic nature of environmental challenges explain the major patterns of deficits accumulation which all groups have observed across the life course.

## Model

### Outline of the model

Let consider the process of environmental challenges imposed by stresses on the organism as a stochastic Poisson process, with intensity rate λ. The average time interval between the consecutive stresses is thus 1/λ. In reality, such challenges are of many different natures, arising from individual exposures to perturbations in the climate, solar activity, weather, pollution, stressful social events, disease outbreaks, etc. The interval between the challenges (1/λ) can span from seconds to months; most cannot be measured. Let *W* be the average time of recovery from such environmental damage in people of the same chronological age. Generally, the time of recovery is also variable, but has a minimum scale of days, spanning up to several months, depending on the nature of the specific damage and the organism’s ability to recover. The ability of the organism to recover depends on the individual’s health or the so-called reserve capacity, itself related to their genetic profile (Christensen et al. 2006; Yashin et al. 2013). Clearly too, an individual’s health is related to the state of the health care in the society, so that not all environmental influence is malign. Despite all this, the time of recovery is age dependent, presumably reflecting subclinical (even microscopic) tissue, cellular and subcellular damage (Howlett and Rockwood 2013).

### Queuing theory and Little’s Law

The schema described above is structurally equivalent to what is well known in stochastic queuing theory—a mathematical discipline that aims to explain how the length of a queue is related to intensity of the steam of arrivals to the system, and to the systems’ priority schedule, and service and waiting times. Queuing theory is widely employed across multiple applications in communications, computer architecture, operation management, to name just a few. The queuing system is usually modeled by a system of differential equations (the Kolmogorov equations). Their structure depends on specific assumptions of the model (e.g., single server or a network of servers; stationary or non-stationary arrivals, different priory schedules, etc.). Despite the great complexity of queuing equations, there is a general and simple relationship between the essential characteristics of queuing systems, known as Little’s Law, which recently celebrated its 50th anniversary (Little 1961, 2011). This law states that *the average number of items in a queuing system (L) equals the average arrival rate (λ) multiplied by the average waiting time of an item in the system, W.*

1 $$ L \, = \lambda W $$

Reformulating Little’s Law in terms of our setting, we suggest that *the average number of deficits present in an individual (L) equals the rate of environmental stresses λ, times the average recovery time W*.

### Deficits accumulate, indicating that recovery time increases with age

**Fig. 1:**
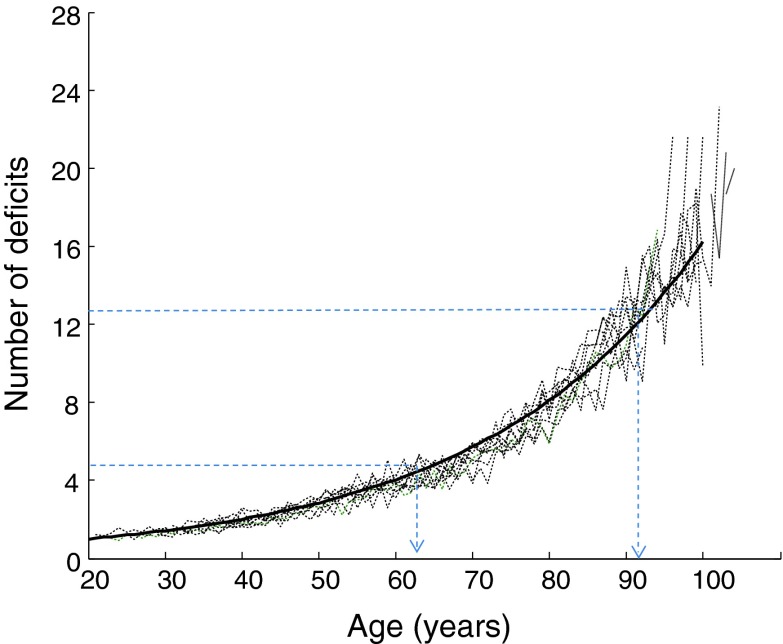
Age trajectories of the mean number of deficits. *Thin lines* are the cross-sectional data for the nine consecutive two-years cycles plotted against age. The *solid line* is the best exponential fit with the exponent of 0.035 (±0.02)

The importance of this relationship is that it states that the average recovery time is *proportional* to the average number of deficits that the individual possesses. Here, the coefficient of proportionality (λ) is a global characteristic of the environment. In general, Little’s Law makes is possible to calculate one of the parameters in Eq. (1) by knowing two others. In some cases, however, even knowing one parameter may help to estimate the second one under the conditions that the third parameter does not change. Here, it suggests that even if during the life course the intensity of the environmental stresses remained constant, the average recovery time would still increase with age. This offers a potentially key insight, i.e. that the kinetics of the deficit accumulation with age is the same as the kinetics of recovery time. The kinetics of deficit accumulation is known to be of an exponential type (Mitnitski et al. 2005; Rockwood et al. 2011) with the exponent parameter typically close to 0.03. This is illustrated in Fig. 1, which shows age-specific (cross-sectional) average trajectories for the 9 waves of the National Population Health Survey of Canadian aged 20+ years, over 16 years of follow-up, repeated every 2 years. Note that the overlaid trajectories virtually coincide and can be fitted with the exponential function with slope 0.035 (±0.02) (the solid line). There is about a threefold increase in the number of deficits during three decades after age 60, and about an order of magnitude increase from age 20 to 100 years. Given the exponential increase in the number of deficits with age, by virtue of Little’s Law we can say that the average recovery time increases exponentially with age, and with the same parameter *k* = 0.035. In other words, the average *W* satisfies the following differential equation:

2 $$ dW/dt \, = \, kW $$

where *k* = *0.035* is the constant slope estimated empirically from the data.

### Changes in the distribution of the number of deficits

**Fig. 2:**
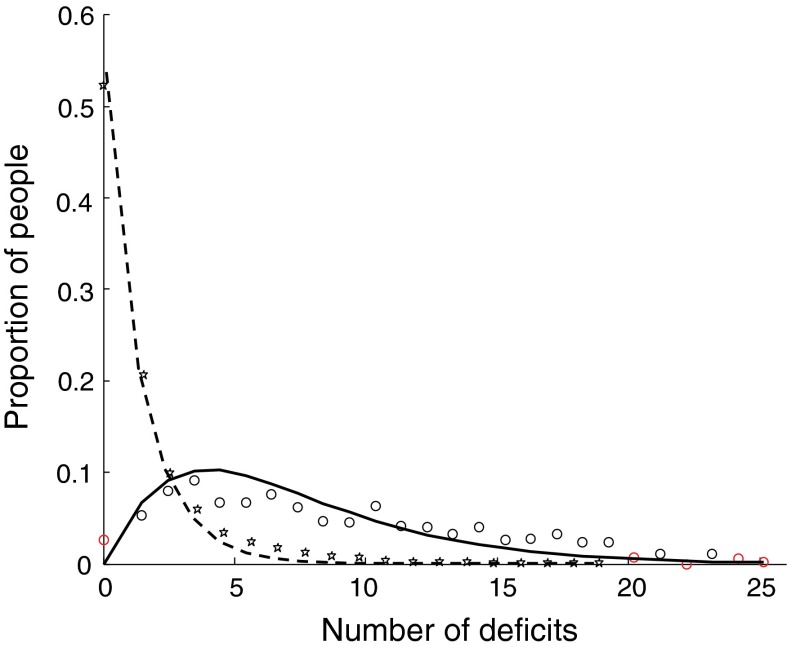
Distribution of the number of deficits for the two age groups: 20–30 years old (*stars* are the observational data and the *dashed line* is the exponential fit with the exponent −0.6) and 75–100 years old (*circles* are the observational data and the *solid line* is the gamma fit with the shape and scale parameters of 2.1 and 3.2, respectively)

Because Little’s Law operates only with the averages, the changes in the distribution of the number of deficits between age groups cannot be seen just from it alone. The patterns of the distribution, however, could be understood from the following simple considerations, consistent with queuing theory. When the recovery time is low (i.e. when the rate of recovery is high), the damage caused by the environmental stresses is quickly recovered, without persisting deficits. This is the case for young people, most of whom have no or a few deficits. This situation is similar to queuing systems with only “light traffic” (or “light utilization”), which show highly asymmetrical density distributions for the length of queue. Indeed, in younger people, the distribution of the number of deficits is highly asymmetrical (Fig. 2 stars and the dashed line for the individuals between 20 and 35 years old). In contrast, when the recovery rate slows, so that recovery time is long, deficits accumulate with age. Likewise, the number of people with zero deficits diminishes. Figure 2 also shows the density distribution of the number of deficits in an older group of individuals aged 75–100 years. The fitting function for the older individuals (the solid line) is well represented by the gamma density function, as is described in “heavy traffic” queuing models (Kyprianou 1972).

## Discussion

In this paper, we have proposed a general and simple stochastic model to explain how the number of deficits present in individuals can be represented by the product of the intensity of environmental stresses to the average recovery time (in accordance with Little’s Law). The exponential increase in the number of health deficits with age directly corresponds to the exponential increase of recovery time, as does the changes with age in the distributions of the deficits. Fast recovery largely prevents deficits from being accumulated, so that the distribution remains highly asymmetrical (most people have no or few deficits). With a decline in the recovery rate, and a corresponding increase of recovery time, fewer people have no deficits and the mode of the distribution shifts to the right. For these reasons, recovery time is the fundamental parameter, which characterizes the health of individuals. Likewise, its population averages even characterize the health of the nations (Theou et al. 2013) as we discuss below.

A large body of evidence can relate an increase in recovery time to age-associated damages and diseases (Franceschi et al. 2000; Rattan 2003; Gurtner et al. 2008; Akushevich et al. 2013; Yanai et al. 2011). The mechanisms by which recovery occurs are complex and involve a number of processes, at different levels of the organism, from the DNA repair response (Moskalev et al. 2012), to repair of chromosomal damage (Nicholls et al. 2011) to autophagy (Couve and Schmachtenberg 2011; Fortini and Dogliotti 2010; Vicencio et al. 2008), degradation of repair capacity (Koga et al. 2011), and a host of others (Tacutu et al. 2010b; Howlett and Rockwood 2013; Yashin et al. 2013). This multiplicity of specific mechanisms responsible for age-related decline in the recovery rate likely corresponds to decline in flexibility (Fabre et al. 2007) or loss of stress resistance with aging seen with decline in allostatic adaptation (Yashin et al. 2007b, c, 2013). The decline in so many specific mechanisms has been characterized as “shrinkage of the homeodynamic space”, due to “the stochastic occurrence and progressive accumulation of molecular damage” (Rattan 2012, 2013).

This age associated increase in recovery time can be regarded as a manifestation of the decline of *vitality* as was postulated by Strehler and Mildvan (1960), although in their model vitality declines linearly whereas the rate of recovery declines exponentially. Decline in the rate of recovery is also in line with the general systems biological mechanism of critical slowing down. Indicators of critical slowing down have been postulated as a means of broadly ranking complex systems, from resilient to fragile (Scheffer 2010; Veraart et al. 2011). We propose that the FI can be considered as such an indicator, in that it indeed provides a means of ranking individuals across grades of fitness and frailty (Rockwood and Mitnitski 2007).

Our model leaves aside the question why the time of recovery increases with age. Being hesitant to answer this question now, we can simply speculate that it reflects an increasing metabolic cost of maintenance (Kirkwood and Holliday 1979; Kirkwood 2011). In general, the entropy of the environment is higher than the entropy of the organism; the latter is far from equilibrium (Nicolis and Prigogine 1979). In consequence, keeping such non-equilibrium conditions is possible for only a limited time (limited by the life span). The incorporation of such a mechanism in the model is a matter for future investigation.

Our results should be interpreted with caution. First, this is a simplistic model, although this is commonly the price paid to make any model tractable. In this regard, there are many ways to make the model more realistic, for example by considering non-Poisson and even non-stationary environments, to allow damage events not be independent of each other. Third, recovery time includes three features that are distinguishable: avoidance of damage by a successful mechanism (which in any case is indistinguishable from instantaneous recovery); resistance (e.g. in a stationary environment, a given exposure such as blunt force trauma might result in bruising in a younger person, but fracture in an older one, which then will require a longer recovery period) and recovery itself. Relatedly, even though Little’s Law holds independently of the priority schedule of dealing with damage, the distributions of the number of deficits will differ for different priority protocols. For example, under heavy traffic conditions (i.e., advanced age) if the strategy of the organism is to deal first with the most important problems, rather than in a way entirely determined by their order of their appearance (i.e. their “arrival”, in queuing terms) this will lead to distributions of deficit number with heavy tails (i.e. more people will have more things wrong). Systems with such priority scheduling recently have been investigated in human dynamics (Barabási 2005; Vázquez et al. 2006) to explain emergence of heavy-tailed distribution in the queues. Also, deficits can emerge in clusters, e.g., more than one deficit might emerge at the same time. This can be addressed under the “multi-server” version of the model, which allows simultaneous acquisition of stresses. The Kolmogorov equations will be different from those for a single server system. Even so, the averages will still follow Little’s Law, which holds independently of the structure of the system. Finally, in contrast to some advanced mathematical models of how health changes during aging (Yashin et al. 2007b, c, 2012, 2013) our model does not include mortality- here we concentrated exclusively on the model being conditional on survival. On the other hand, the number of deficits has proven to be a strong predictor of mortality, and always superior in this regard to chronological age (Mitnitski et al. 2005; Kulminski et al. 2007). For these reasons, we believe that considering these processes separately seems a good approximation of a more general process that includes longitudinal changes in deficits accumulation and mortality.

Despite these limitations, our model makes clear why and how health deficits accumulate with age, providing a systemic mechanism underlying the origin of biological ageing. The model captures the stochastic nature of the environmental stresses that cause damage (Gavrilov and Gavrilova 2006) and shows how an increase in recovery time explains the major patterns of deficit accumulation with aging we so far observed in all datasets. The model can be fine-tuned as new data on biological markers or performance measures become more widely available. In this way, it can organize a framework for better understanding now hidden mechanisms of how health deteriorates during aging.

Our model explains not only the major patterns in deficit accumulation in individuals but also clarifies differences in deficit accumulation between countries with different socioeconomic conditions. The average time of recovery at the level of population depends on the health care system and the latter is related to health care expenditures, as recently demonstrated by Theou et al. (2013). That study reported on more than 36,000 community dwelling people aged 50 and older from 15 European countries. In higher income countries, people had about 3 fewer deficits than in lower-income countries; similarly, the deficit number was negatively correlated with both gross domestic product (r = − 0.79) and health expenditure (r = − 0.63) (Theou et al. 2013). A finding of a similar wealth-health gradient was reported by Semyonov et al. (2013).

The number of deficits individuals accumulate can be modifiable either by medical interventions, changes in the lifestyle, diet and exercise- all these factors are associated with the life span extension because the number of deficits diminishes in the individual who make such changes (Woo et al. 2006; Hubbard et al. 2009a, b; Fallah et al. 2009) and as it becomes clear, because they reduce the time of recovery.

## Conclusions

The age associated increase in recovery time results in the accumulation of deficits as people grow older. This framework not only explains why the number of deficits can be used to estimate individual differences in aging rates but also suggests that targeting the recovery rate should decrease the number of deficits that individuals accumulate, and thereby improve life expectancy.

## Electronic supplementary material

Below is the link to the electronic supplementary material.

Supplementary material 1 (DOC 23 kb)
