# Supplementary material for: Assessing biological aging: the origin of deficit accumulation
Source: Biogerontology. 2013 Jul 17;14(6):709–17. doi: 10.1007/s10522-013-9446-3 (PMC3847281; doi:10.1007/s10522-013-9446-3)
Supplement: Supplementary file 1 — Supplementary material 1 (DOC 23 kb) [file 10522_2013_9446_MOESM1_ESM.doc]

Appendix. Supplementary file

*Subjects and setting*

We analyzed data from the National Population Health Survey, (NPHS) a large Canadian study conducted in seven waves from 1994 to 2010 (n=13,458 at baseline) from age 20 years. The NPHS contains longitudinal information on the health of Canadians and has been the subject of other reports by our group (Mitnitski et al. 2007b; Song et al. 2010; Rockwood et al. 2011). Self-reported information was gathered at baseline and every two years, as was vital status.

*Deficits count (frailty index, FI)*

Thirty-nine dichotomized variables (referred to as “deficits”) including medical conditions, disabilities and health history (see for example the supplementary materials to Rockwood et al., 2010, <http://www.cmaj.ca/content/suppl/2011/04/26/cmaj.101271.DC1/change-rockwood-2-at.pdf> ) were used to calculate each individual’s health status, combined in a frailty index (FI). Data were coded so that the presence of a deficit was represented by a “1”, and the absence of the deficit by “0”. For any individual, their frailty index represents the number of deficits present, divided by 39 (the number of deficits considered). Note that the theoretical range of the FI is 0 (no deficits) to 1 (all 39 deficits present). Note too that individuals with a score of 0 are said to belong to the “zero state” and are considered to be the fittest. The FI represents health of the individuals on the fitness-frailty scale from the fittest with 0 deficits to theoretically 1 (all deficits are present). Of note, none of the participants of the study has more than 27 deficits (70%) of the total number of deficits available.
